# Evaluating a Geriatric Educational Program: Exploring Opportunities for Increasing Impact and Scale

**DOI:** 10.1093/geroni/igab046.2793

**Published:** 2021-12-17

**Authors:** Shera Hosseini, Michelle Howard, Allison Ward

**Affiliations:** McMaster University, Hamilton, Ontario, Canada

## Abstract

The geriatric population is rapidly growing, and this growth is beyond the pace of increase in the number of healthcare professionals who are qualified to care for and tend to the various needs of this significant subgroup of the population. The current university curricula have not been sufficient in terms of quantity as well as their ability to address the ageism inherent in the perspectives of students from across the educational spectrum. In recognition of the absence of standardized geriatric guidelines, medical associations across Canada and the United States have established geriatric learning competencies for medical programs. Nevertheless, there are exiting gaps regarding the development and evaluation of geriatric-focused didactic programs that adequately train and build competency among the students interested in pursuing careers with geriatric-specific elements. A university-wide program was developed to enhance aging education and build competency through sparking interest, providing better education related to aging, and building better relationships between future healthcare professionals and older adults. To evaluate the impact of this program, a logical framework was developed a-priori and revised through constant iterations and following discussion with the program’s multidisciplinary stakeholder group. Quantitative measures are being augmented with in-depth qualitative interviews to explore elements influencing students’ experiences with the program and the effect on their interests in and attitudes towards geriatrics. The results will inform our conclusions regarding program effectiveness in enhancing interest in geriatric-focused education among the students and trainees and assist with recommending future directions regarding impact and large-scale dissemination and implementation.

